# Comparative Diagnostic Performance of RDW-to-Albumin and CRP-to-Albumin Ratios in Neonatal Sepsis: A Retrospective Cohort Study

**DOI:** 10.3390/diagnostics16182942

**Published:** 2026-09-11

**Authors:** Filiz Aktürk Acar, Yakup Aslan, Mehmet Mutlu, Zeliha Aydın Kasap, Şebnem Kader, Gülçin Bayramoğlu

**Affiliations:** 1 Department of Neonatology, Faculty of Medicine, Karadeniz Technical University, Trabzon 61080, Türkiye; 2 Department of Neonatology, Faculty of Medicine, Erciyes University, Kayseri 38039, Türkiye; 3 Department of Biostatistics and Medical Informatics, Faculty of Medicine, Karadeniz Technical University, Trabzon 61080, Türkiye; 4 Department of Neonatology, Kırklareli Training and Research Hospital, Kirklareli 39010, Türkiye; 5 Department of Medical Microbiology, Faculty of Medicine, Karadeniz Technical University, Trabzon 61080, Türkiye

**Keywords:** red cell distribution width, albumin, RAR, CAR, neonatal sepsis, composite indices

## Abstract

**Background/Objectives:** Neonatal sepsis remains a major cause of morbidity and mortality worldwide, underscoring the critical need for early and accurate diagnostic biomarkers. This study aimed to evaluate the predictive value of the red cell distribution width-to-albumin ratio (RAR) for the diagnosis of neonatal sepsis and to compare its diagnostic performance with the C-reactive protein-to-albumin ratio (CAR) and other hematological parameters. **Methods:** This retrospective cohort study was conducted in the Neonatology Department of Karadeniz Technical University Faculty of Medicine between January 2015 and June 2025. A total of 874 neonates with a gestational age > 36 weeks were included: 411 with blood culture-confirmed sepsis (108 Gram-negative, 303 Gram-positive) and 463 healthy controls. Diagnostic performance was assessed using receiver operating characteristic (ROC) curve analysis, with optimal cut-off values determined by the Youden index. **Results:** RAR demonstrated modest diagnostic accuracy for neonatal sepsis, with an area under the curve (AUC) of 0.647 (95% CI: 0.61–0.69), a cut-off value of 5.108, sensitivity of 50.7%, and specificity of 76.7%. RAR was significantly elevated in the Gram-negative group compared with Gram-positive cases and controls (*p* < 0.001). However, the clinical significance of this subgroup difference requires further investigation. In multivariable logistic regression analysis, RAR remained independently associated with culture-confirmed neonatal sepsis after adjustment for gestational age, birth weight, sex, and mode of delivery (aOR = 2.190, 95% CI: 1.838–2.608, *p* < 0.001). Among all evaluated markers, CAR achieved the highest diagnostic performance (AUC: 0.981, sensitivity: 88.5%, specificity: 98.7%, PPV: 98.4%, NPV: 90.7%, LR+: 68.47), followed by C-reactive protein (AUC: 0.978), while neutrophil-to-lymphocyte ratio showed the lowest discriminatory ability (AUC: 0.592). **Conclusions:** RAR showed limited standalone discriminatory performance for culture-confirmed neonatal sepsis, although its association with sepsis remained significant after adjustment for relevant demographic and perinatal factors. CAR demonstrated significantly greater discriminatory performance than RAR. Further studies are needed to determine whether RAR provides incremental value when incorporated into multivariable diagnostic models.

## 1. Introduction

Neonatal sepsis remains a leading cause of morbidity and mortality in newborns worldwide, despite technological and clinical advances in neonatology [1]. Neonatal clinical symptoms are often non-specific and may overlap with non-infectious conditions, making sepsis difficult to distinguish based solely on clinical presentation [1,2,3]. Perinatal factors such as nuchal cord have also been shown to influence early neonatal clinical assessment, further complicating clinical differentiation [4]. Delayed diagnosis can result in rapid clinical deterioration and death, while overdiagnosis leads to unnecessary antibiotic exposure, contributing to antimicrobial resistance [5] and disruption of the neonatal microbiome [6].

Blood culture remains the gold standard for confirming neonatal sepsis; however, it has well-recognized limitations, including extended result times ranging from hours to several days [6,7,8] and the possibility of false-negative results due to various reasons (inadequate blood volume, early sampling, collection after antibiotic initiation, or infection by fastidious organisms) [1]. These limitations underscore the urgent need for rapid, reliable, and cost-effective biomarkers that can support early clinical decision-making.

C-reactive protein (CRP) is among the most widely used inflammatory markers in neonatal sepsis, yet its diagnostic utility is limited by low specificity. CRP levels can be elevated in non-infectious conditions such as perinatal asphyxia, intraventricular hemorrhage, meconium aspiration syndrome, and therapeutic hypothermia, which are all common in the neonatal period [9,10,11]. Similarly, serum albumin exhibits anti-inflammatory properties, and its levels are known to decrease inversely with disease severity [12]. The degree of hypoalbuminemia has been shown to correlate directly with sepsis severity [13,14]. In recent years, the CRP-albumin ratio (CAR) has emerged as a more comprehensive composite index. Several studies have evaluated the diagnostic value of CAR in neonatal sepsis and determined that it has good discriminatory power in predicting sepsis [6,15,16].

Red blood cell distribution width (RDW), a parameter automatically derived from routine complete blood count panels that reflects the distribution of red blood cell volumes, has been shown in various studies to be a diagnostic and prognostic marker in neonatal sepsis [3,17,18,19]. In sepsis, the acute systemic inflammatory response causes an increase in RDW levels [17]. The RDW-to-albumin ratio (RAR) is a novel composite index that is based on both RDW and albumin levels. Previous studies have evaluated RAR mostly in adult patients and reported it as a useful diagnostic and prognostic indicator for inflammatory conditions in adult populations, including sepsis [12,13,20,21], diabetes mellitus [22], and cardiovascular diseases [23]. Furthermore, a recent large pediatric cohort study demonstrated that RAR was independently associated with adverse clinical outcomes in critically ill children [24]. However, to the best of our knowledge, no study to date has evaluated the diagnostic utility of RAR specifically in neonatal sepsis.

In this retrospective cohort study, we aimed to evaluate the diagnostic performance of RAR for culture-confirmed neonatal sepsis and to compare it with CAR and other routinely available hematological and biochemical parameters. We further investigated whether RAR could differentiate Gram-negative from Gram-positive sepsis, given the distinct pathophysiological responses elicited by these two pathogen groups. We hypothesized that RAR would be elevated in neonates with sepsis and investigated whether its levels differed according to microbiological subgroup.

## 2. Materials and Methods

### 2.1. Study Design and Setting

This retrospective cohort study was conducted at the Department of Neonatology, Faculty of Medicine, Karadeniz Technical University, Trabzon, Türkiye, between 2015 and 2025. Ethical approval was obtained from the institutional ethics committee (Protocol No: 2025/225; Date: 15 September 2025). As the study was retrospective in design, informed consent was not required. All data were fully anonymized, and the study was conducted in accordance with the principles of the Declaration of Helsinki. The manuscript was prepared in accordance with the STROBE (Strengthening the Reporting of Observational Studies in Epidemiology) reporting guidelines [25].

### 2.2. Study Population and Inclusion/Exclusion Criteria

Neonates with a gestational age greater than 36 weeks who were diagnosed with sepsis confirmed by positive blood culture were included in the study. The control group comprised neonates without a diagnosis of sepsis and with negative CRP and procalcitonin values, who were managed in one of three clinical settings: the NICU (for non-infectious conditions such as neonatal jaundice, respiratory distress, or feeding difficulties), the postnatal ward, or the well-child outpatient clinic. Patients were identified through a retrospective review of the hospital’s electronic medical records system and laboratory database.

Inclusion criteria: (1) being in the neonatal age group, (2) gestational age greater than 36 weeks, (3) positive blood culture in the neonatal period. For coagulase-negative staphylococci (CoNS) and other potential skin contaminants, growth of the same microorganism in two separate blood cultures was required for the isolate to be considered clinically significant. (4) Availability of complete demographic and laboratory data records. For the control group, neonates with a gestational age greater than 36 weeks who had no clinical or laboratory findings suggestive of sepsis were included.

Exclusion criteria: (1) postnatal age greater than 28 days at the time of diagnosis, (2) gestational age of 36 weeks or less (preterm neonates), (3) any condition known to cause non-infectious CRP elevation or albumin alteration (e.g., perinatal asphyxia, cholestasis), (4) neonates with a clinical diagnosis of sepsis but without a confirmed positive blood culture, (5) CoNS and other potential skin contaminants isolated in only a single blood culture, (6) neonates with fungal organism isolated in blood culture, (7) multiple congenital anomalies, (8) hematologic diseases, and (9) incomplete or missing medical records.

### 2.3. Data Collection and Measurements

The following data were retrospectively collected from hospital records:

- Demographic and clinical characteristics;
- Laboratory results: Complete blood count parameters, including white blood cell count (WBC), red blood cell (RBC), hemoglobin (Hb), hematocrit (Htc), RDW, platelet (PLT), neutrophil and lymphocyte count; Biochemical parameters, including serum albumin, CRP, and procalcitonin; microbiological data, including blood culture results and identified pathogens;
- Composite inflammatory indices: RAR, CAR, neutrophil-to-lymphocyte ratio (NLR), platelet-to-albumin ratio (PAR), and platelet-to-lymphocyte ratio (PLR).

Complete blood count and biochemical parameters were recorded at the time of sepsis diagnosis in the sepsis group. Blood samples for culture and all laboratory biomarker measurements were collected simultaneously prior to the initiation of empirical antibiotic therapy in all cases, in accordance with the standard clinical protocol of our NICU. In the control group, the corresponding values were obtained at a matched postnatal age to ensure comparability between the groups.

Complete blood count parameters were obtained using the Mindray BC 6200 hematology analyzer (Mindray, Shenzhen, China). Biochemical parameters, including CRP and albumin, were measured using the Beckman Coulter AU 5800 autoanalyzer (Beckman Coulter, Shizuoka, Japan). Procalcitonin levels were measured using the Abbott Alinity i A1 analyzer (Abbott, Singapore).

### 2.4. Dataset and Study Outcomes

As the study data were collected retrospectively, eligible cases were identified using a non-probability consecutive sampling method.

The primary outcome of this study was to evaluate the diagnostic performance of the RAR in predicting neonatal sepsis, assessed by receiver operating characteristic (ROC) curve analysis, including the area under the curve (AUC), sensitivity, specificity, and optimal cut-off value. The secondary outcome was to compare the diagnostic accuracy of RAR with that of the CAR in the same study population, in order to determine the superior inflammatory composite indices for the identification of neonatal sepsis.

### 2.5. Statistical Analysis

Descriptive statistics for continuous variables are presented as mean ± standard deviation for normally distributed data and as median (Q1–Q3) for non-normally distributed data. Categorical variables are presented as frequency and percentage. The normality of continuous variables was assessed using the Kolmogorov–Smirnov test. Missing data were assessed for all laboratory variables. Data regarding CRP were missing in 19 of 874 neonates (2.2%), while data for each of MPV and PDW were missing in 5 neonates (0.6%). Missing values for these variables were imputed using the median of the respective variable. No missing observations were present for the other laboratory variables included in the analyses. Procalcitonin values were not applicable in 503 of 874 neonates (57.6%), and therefore they were excluded from further analyses. Differences between two independent groups were evaluated using Student’s *t*-test for normally distributed continuous variables and the Mann–Whitney U test for non-normally distributed continuous variables; categorical variables were compared using the Chi-square test. For comparisons among the three groups (Gram-negative, Gram-positive, and control), the Kruskal–Wallis test was applied, with post hoc pairwise comparisons performed using Dunn’s test with Bonferroni correction. The association between mode of delivery and group distribution was examined using the Pearson Chi-square test; adjusted residuals were used to identify cell-level differences, with |z| > 1.96 considered statistically significant.

A multivariable binary logistic regression analysis was performed to assess whether RAR was independently associated with culture-confirmed neonatal sepsis after adjustment for potential confounding factors. Sepsis status (1 = sepsis, 0 = non-sepsis/control) was entered as the dependent variable, while RAR, gestational age, birth weight, sex, and mode of delivery were entered simultaneously as independent variables. Birth weight was entered in 100-g units. Adjusted odds ratios (aORs) with 95% confidence intervals (CIs) were reported. Model fit was assessed using the Hosmer–Lemeshow goodness-of-fit test and Nagelkerke R^2^.

Receiver operating characteristic (ROC) curve analysis was performed to evaluate the diagnostic performance of all assessed biomarkers (RAR, CAR, CRP, albumin, NLR, RBC, and Hb) for neonatal sepsis, and optimal cut-off values were determined using the Youden index. For biomarkers for which lower values are associated with neonatal sepsis (albumin, RBC, and Hb), values were multiplied by −1 prior to ROC analysis to ensure consistent curve directionality. The AUCs of CAR and RAR were compared using DeLong’s test for two correlated ROC curves. The AUCs of CAR and CRP were also directly compared using the paired DeLong test. Statistical significance was defined as a two-sided *p*-value < 0.05 for all analyses. Descriptive and comparative statistical analyses were performed using IBM SPSS Statistics version 23 (IBM Corp., Armonk, NY, USA; licensed to Karadeniz Technical University, Trabzon, Türkiye). Data visualization was performed in R version 4.3 (R Foundation for Statistical Computing, Vienna, Austria) using the pROC, ggplot2, and GGally packages.

## 3. Results

### 3.1. Study Population and Demographic Characteristics

**Figure 1:**
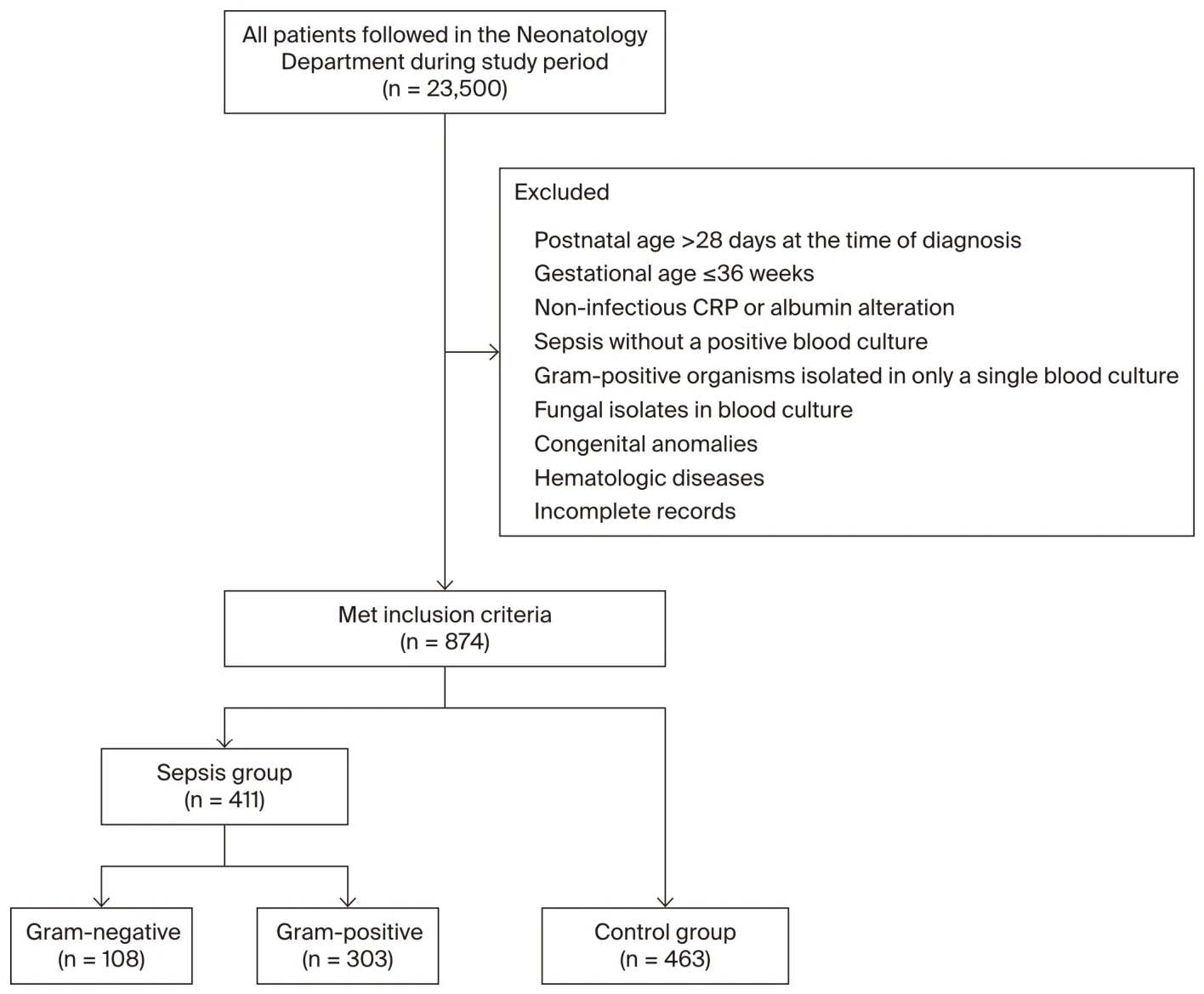
Patient flow chart showing the selection and distribution of the study cohort.

As shown in Figure 1, a total of 874 neonates with a gestational age of 37–41 weeks were enrolled in the study, of whom 411 (47%) had blood culture-confirmed sepsis and 463 (53%) were healthy controls. Among the sepsis group, 303 (73.7%) had Gram-positive and 108 (26.3%) had Gram-negative infections. Among the 303 Gram-positive infections, definite CoNS accounted for 232 cases (76.6%). The most frequent Gram-positive isolates were *Staphylococcus epidermidis* (*n* = 147), *Staphylococcus haemolyticus* (*n* = 39), *Staphylococcus aureus* (*n* = 29), and *Staphylococcus hominis* (*n* = 27). Among the 108 Gram-negative infections, the most frequent isolates were *Klebsiella pneumoniae* (*n* = 20), *Enterobacter cloacae* (*n* = 15), and *Escherichia coli* (*n* = 13).

Gestational age differed significantly across the three groups (*p* = 0.001), with post hoc analysis revealing a significant difference between the Gram-positive and control groups (*p* < 0.05), while no significant difference was observed between the Gram-negative and control groups. Birth weight and sex distribution did not differ significantly among the groups (*p* = 0.499 and *p* = 0.575, respectively). Demographic characteristics are summarized in Table 1.

### 3.2. Comparison of Hematological and Biochemical Parameters

Comparison of hematological and biochemical parameters across the three groups is presented in Table 2. Procalcitonin was excluded from the analysis due to missing data in more than 60% of cases.

When the sepsis and non-sepsis groups were compared, WBC, RBC, hemoglobin, RDW, platelet count, albumin, CRP, RAR, CAR, and NLR showed statistically significant differences between the two groups (*p* < 0.05).

The median CRP level in the overall sepsis group was 1.70 mg/dL (IQR: 0.70–3.10), whereas it was 0.04 mg/dL (IQR: 0.02–0.12) in the non-sepsis group. RBC count, hemoglobin, and albumin levels were significantly lower in the sepsis group compared to the control group, while RDW, RAR, CAR, NLR, and PLR were significantly elevated in the sepsis group. In contrast, no statistically significant difference was found between the groups in terms of PAR (*p* > 0.05).

### 3.3. Gram-Negative vs. Gram-Positive Group Comparison

Significant differences were observed between the Gram-negative and Gram-positive groups across several parameters. WBC was significantly lower in the Gram-negative group compared to the Gram-positive group (*p* < 0.001). RBC count, hemoglobin, and platelet count were also significantly lower in Gram-negative cases (*p* < 0.001 for all). Serum albumin followed an inverse pattern with inflammation severity: lowest in the Gram-negative group (median 3.05 g/dL), intermediate in the Gram-positive group (3.40 g/dL), and highest in the control group (3.50 g/dL), with all pairwise comparisons reaching statistical significance.

RAR was significantly elevated in the Gram-negative group compared to both the Gram-positive group and controls (*p* < 0.001), with all pairwise comparisons being statistically significant. Similarly, CRP and CAR were highest in the Gram-negative group, consistent with a more pronounced systemic inflammatory response elicited by Gram-negative pathogens. RDW alone did not differ significantly across the Gram-negative and Gram-positive groups (*p* = 0.552).

In ROC analysis restricted to the 411 neonates with culture-confirmed sepsis, RAR showed limited discrimination between Gram-negative and Gram-positive infections (AUC = 0.621, 95% CI: 0.559–0.683). RDW showed essentially no discriminatory ability (AUC = 0.543, 95% CI: 0.476–0.610), whereas albumin showed greater discrimination (AUC = 0.674, 95% CI: 0.614–0.734). Albumin had a significantly higher AUC than RAR (*p* = 0.004), whereas the difference between RAR and RDW was not significant (*p* = 0.186).

### 3.4. Diagnostic Performance—ROC Curve Analysis

**Figure 2:**
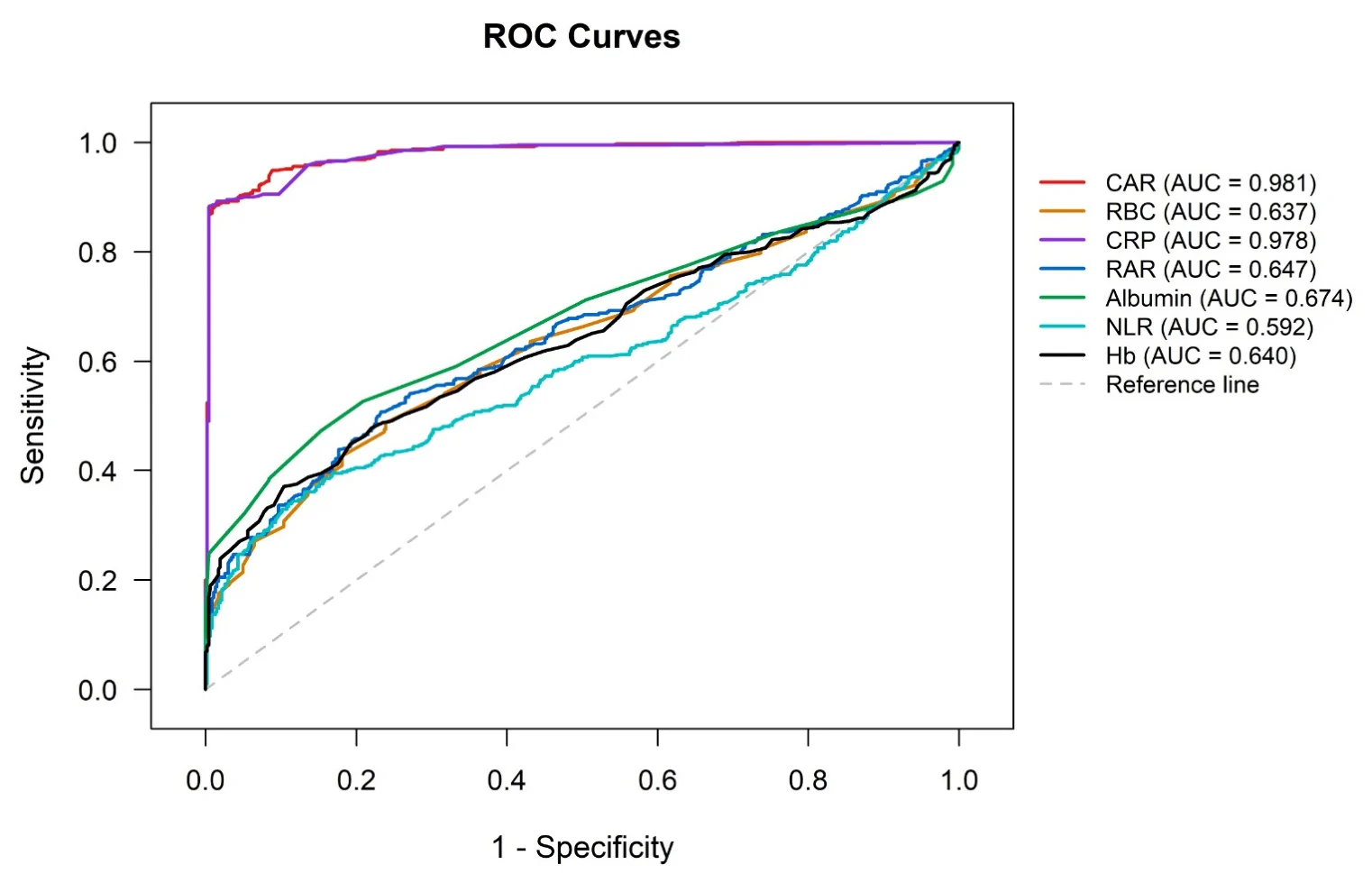
Receiver operating characteristic (ROC) curves for the evaluated biomarkers in the diagnosis of bacterial sepsis. The area under the curve (AUC) values are presented in parentheses. CAR (C-reactive protein-to-albumin ratio) and CRP (C-reactive protein) demonstrated the highest discriminatory performance with AUC values of 0.981 and 0.978, respectively. RAR (red cell distribution width-to-albumin ratio), Albumin, NLR (neutrophil-to-lymphocyte ratio), RBC (red blood cell count), and Hb (hemoglobin) showed moderate discriminatory ability with AUC values ranging from 0.592 to 0.674. For inverse biomarkers (Albumin, RBC, and Hb), values were multiplied by −1 prior to analysis to ensure consistent directionality across all ROC curves. The gray dashed diagonal line represents the reference line.

ROC curve analysis was performed for all evaluated biomarkers; results are presented in Table 3 and illustrated in Figure 2. For biomarkers where lower values are associated with neonatal sepsis (albumin, RBC, hemoglobin), values were multiplied by −1 prior to analysis to ensure consistent curve directionality.

CAR demonstrated markedly greater discriminatory performance than RAR, with AUCs of 0.981 and 0.647, respectively. Paired comparison of the two correlated ROC curves using DeLong’s test confirmed that this difference was statistically significant (ΔAUC = 0.333, 95% CI: 0.296–0.370; Z = 17.546, *p* < 0.001). Direct comparison of CAR and CRP showed a statistically significant but very small difference in AUC (0.981 vs. 0.978; ΔAUC = 0.0024, 95% CI: 0.0005–0.0042; Z = 2.486, *p* = 0.013).

RAR achieved an AUC of 0.647 (95% CI: 0.61–0.69) at a cut-off of 5.108, with a sensitivity of 50.7%, specificity of 76.7%, PPV of 65.8%, NPV of 63.8%, and a Youden index of 0.28. Albumin yielded an AUC of 0.674, with a specificity of 84.7% at a threshold of ≤3.25 g/dL. NLR showed the lowest discriminatory ability among the evaluated markers (AUC: 0.592). WBC, PLR, RDW, and PAR were excluded from the ROC figure due to AUC values ranging from 0.508 to 0.553, indicating performance not meaningfully above chance.

Bootstrap out-of-bag validation yielded internally evaluated sensitivity/specificity estimates of 48.6%/76.4% for RAR, 89.2%/96.8% for CAR, 88.5%/99.0% for CRP, 35.2%/86.5% for NLR, and 47.9%/82.8% for albumin.

Multivariable binary logistic regression analysis was performed to assess whether the association between RAR and culture-confirmed neonatal sepsis persisted after adjustment for potential confounders, such as gestational age, birth weight, sex, and mode of delivery. RAR remained independently associated with culture-confirmed neonatal sepsis (aOR = 2.190, 95% CI: 1.838–2.608, *p* < 0.001). Gestational age was also independently associated with sepsis (aOR = 1.271, 95% CI: 1.096–1.474, *p* = 0.002), whereas birth weight (aOR = 0.983 per 100-g increase, 95% CI: 0.952–1.016, *p* = 0.309) and sex (aOR = 1.158, 95% CI: 0.860–1.561, *p* = 0.334) were not statistically significant. Mode of delivery was significantly associated with sepsis; compared with NSVD, cesarean delivery was associated with lower odds of sepsis (aOR = 0.278, 95% CI: 0.193–0.401, *p* < 0.001).

The overall model was statistically significant (likelihood-ratio χ^2^(5) = 167.709, *p* < 0.001) and showed adequate goodness of fit (Hosmer–Lemeshow χ^2^(8) = 2.427, *p* = 0.965), with a Nagelkerke R^2^ of 0.233. Overall classification accuracy was 68.5% at a probability cut-off of 0.50 (Table 4).

## 4. Discussion

In this study, the diagnostic and discriminatory value of RAR was evaluated in neonates with blood culture-confirmed sepsis and compared with the CAR and other hematological parameters. To the best of our knowledge, this is the first study to evaluate the diagnostic utility of RAR in neonates with blood culture-confirmed sepsis.

Our findings showed that RAR had limited standalone discriminatory performance for neonatal sepsis. Although RAR levels were significantly higher in the Gram-negative group than in the Gram-positive and control groups, the clinical significance of this difference and its potential utility for distinguishing microbiological subgroups require further investigation. In contrast, CAR demonstrated substantially greater discriminatory performance than RAR in this cohort.

When we examine the components of these composite indices separately, it is seen that RDW, which constitutes the share of RAR, also has value as an independent inflammatory marker in sepsis. Although blood culture is the gold standard for the diagnosis of sepsis, the inherent delay in obtaining results and the non-specific signs and symptoms of sepsis in neonates continue to underscore the need for reliable and readily available biomarkers for early diagnosis. In this regard, numerous studies have examined the role of RDW in the context of sepsis in both adult and neonatal populations [3,18,26,27]. In sepsis, RDW has been shown to increase in association with systemic inflammation and oxidative stress-mediated disruption of erythropoiesis [3,24]. Bulut et al. [18] showed that RDW values were significantly higher in neonatal cases with sepsis compared to non-sepsis cases, and its diagnostic performance in ROC curve analysis was even higher than CRP. Similarly, Hu et al. [26] reported that increased RDW was associated with short- and long-term adverse outcomes in both adult and neonatal sepsis. In our study, RDW was also found to be significantly higher in sepsis patients compared to the control group (*p* < 0.05); however, this statistical significance was not maintained in the three-group comparison (*p* > 0.05).

Another widely used inflammatory marker in the diagnosis of sepsis is CRP. However, its diagnostic utility is limited by low specificity, particularly due to the physiological rise observed after birth and in non-infectious conditions [9,10,11]. Non-infectious perinatal conditions may present with clinical and laboratory findings that overlap with those of early-onset sepsis, highlighting the need to interpret inflammatory biomarkers within the clinical context [28]. Single measurements of CRP and procalcitonin have limited diagnostic accuracy; serial measurements may be more useful in supporting the exclusion of sepsis when interpreted alongside clinical findings and blood culture results [1]. Albumin, another inflammatory marker, is a negative acute phase reactant whose levels rapidly decrease in systemic inflammation due to increased vascular permeability, reduced hepatic synthesis, and accelerated catabolism. During sepsis, the decrease in albumin not only reflects the severity of the inflammatory response but also leads to adverse hemodynamic outcomes such as hypotension and hypovolemia due to changes in oncotic pressure [6]. Previous studies have shown that low serum albumin levels are associated with sepsis severity and poor prognostic outcomes [13,14]. In the present study, both CRP and albumin levels differed significantly between the sepsis and control groups (*p* < 0.05), further confirming the established roles of these markers in the inflammatory cascade of sepsis.

Other inflammatory markers used in the diagnosis of sepsis are composite indices such as RAR and CAR, which are obtained by dividing RDW and CRP by albumin. Given that albumin constitutes the denominator of both RAR and CAR, its significant reduction in the sepsis group likely contributed to the elevation of these composite indices, thereby enhancing their discriminative capacity. Several studies in the literature have evaluated the diagnostic utility of CAR in neonatal sepsis [6,15,16]. In a study conducted by Yang et al. [15] involving 214 premature neonates, the utility of the high-sensitivity CRP (hs-CRP)/albumin ratio was investigated in premature infants with early-onset intrauterine infection, and the 48-h hs-CRP/albumin ratio was found to be significantly elevated in the infection group compared to the non-infection group. ROC curve analysis revealed an AUC of 0.765, with a sensitivity of 84.2% and a specificity of 76.3% at a cut-off value of 0.08, suggesting that the hs-CRP/albumin ratio measured at 48 h after NICU admission may enhance the sensitivity of early infection diagnosis in premature neonates. Li et al. [6] reported that CAR levels were significantly elevated in neonates with sepsis and demonstrated a stepwise increase from the control group through mild to severe sepsis. In the same study, ROC curve analysis revealed an AUC of 0.74, indicating that CAR possesses good discriminatory power in predicting neonatal sepsis. In another study by Güneş et al. [16], CAR was identified as a promising biomarker for Gram-negative bacteremia in late-onset neonatal sepsis. In that study, CAR was evaluated in 60 preterm neonates with positive blood cultures—selected from a cohort of 112 neonates diagnosed with sepsis—and was found to be significantly elevated in the Gram-negative sepsis group. ROC curve analysis demonstrated that the optimal cut-off value of CAR for the prediction of Gram-negative sepsis was >35.17, yielding a specificity of 97% and a sensitivity of 56% (AUC = 0.839; 95% CI: 0.743–0.944; *p* < 0.001). In the present study, CAR demonstrated markedly superior diagnostic performance compared to RAR, showing high sensitivity and specificity with an AUC of 0.98, further consolidating the evidence for the diagnostic utility of CRP–albumin-based composite indices in culture-confirmed neonatal sepsis.

In contrast, RAR has been predominantly investigated in adult inflammatory conditions [12,13,20,21], and to the best of our knowledge, no prior study has examined its diagnostic value specifically in neonatal sepsis. A recent retrospective cohort study by Jing et al. [24], conducted in a large pediatric intensive care unit (PICU) cohort of 7075 patients, supported the prognostic significance of RAR in critically ill pediatric patients, demonstrating that higher RAR values were independently associated with increased 28-day, 90-day, and in-hospital mortality rates. It should be noted, however, that only 1919 patients (27%) in that cohort had a diagnosis of bacteremia, and the relationship between RAR and sepsis diagnosis was not specifically evaluated. Örnek et al. [29] evaluated the prognostic value of RAR in a cohort of 1151 children followed in PICU due to community-acquired pneumonia; it was found that RAR performed better than its own components (RDW and albumin) in predicting adverse clinical outcomes, and no other parameter surpassed RAR in terms of predictive performance in ROC analyses. Although RAR differed between Gram-negative and Gram-positive sepsis, its discriminatory performance between these groups was limited. Albumin showed greater discriminatory performance than RAR, whereas RDW showed essentially no discriminatory ability, suggesting that the observed RAR difference may be predominantly related to its albumin component. Based on these findings, the present study extends the evaluation of RAR to neonates with culture-confirmed sepsis. Although RAR differed significantly between neonates with and without sepsis, its standalone discriminatory performance was limited (AUC = 0.647). Nevertheless, RAR remained independently associated with culture-confirmed neonatal sepsis after adjustment for gestational age, birth weight, sex, and mode of delivery. RAR levels were also significantly higher in the Gram-negative subgroup than in the Gram-positive and control groups; however, whether this difference has clinical utility for distinguishing pathogen groups requires further investigation.

The limitations of this study include its retrospective, single-center design, and the exclusion of procalcitonin data from the analysis due to a high rate of missing values. Neonatal severity scores such as Apgar and SNAPPE-II were not included in the study design from the outset. Because CRP and procalcitonin results formed part of the retrospective clinical information used in defining the non-septic control group, selection/incorporation bias cannot be excluded, particularly for CRP and CAR. This may have contributed to the very high AUC estimates observed for these markers. In addition, the control cohort included neonates from different clinical settings, and reliable patient-level retrospective classification into symptomatic non-septic and healthy control subgroups was not available in the analytical dataset. Therefore, spectrum bias cannot be excluded, and the diagnostic performance estimates should be interpreted cautiously. This represents an additional limitation of the study.

However, although CAR had a statistically higher AUC than CRP in the paired DeLong comparison, the absolute difference was very small (ΔAUC = 0.0024), and the present findings therefore do not establish a substantial incremental diagnostic benefit of adding albumin to CRP.

In conclusion, RAR showed limited standalone discriminatory performance for culture-confirmed neonatal sepsis and therefore cannot be considered a sufficiently accurate standalone diagnostic biomarker based on the present findings. Nevertheless, RAR remained independently associated with neonatal sepsis after adjustment for gestational age, birth weight, sex, and mode of delivery. In contrast, CAR demonstrated significantly greater discriminatory performance than RAR. Although RAR may reflect sepsis-associated hematological and inflammatory alterations, its potential incremental value within multivariable clinical or biomarker-based models remains to be established. Further prospective, multicenter studies are warranted to determine whether RAR provides additional clinical value beyond established biomarkers.

## Figures and Tables

**Table 1:** Demographic characteristics of groups.

	Sepsis (*n* = 411)		Non-Sepsis (*n* = 463)		
Variables	Gram Negative (*n* = 108) Group 1	Gram Positive (*n* = 303) Group 2	Control Group (*n* = 463) Group 3	*p* (Three Group Comparisons)	*p* (Sepsis–Non- Sepsis)
GA, weeks	38 (37–39)	38 (37–40)	38 (37–39)	0.001 *	0.009 ^#^
BW, g	3106.81 ± 622.35	3170.45 ± 503.56	3163.88 ± 460.89	0.499 **	0.604 ^##^
Sex, *n* (%)					
Female	45 (11.8)	126 (33.2)	209 (55.0)	0.575 ***	0.274 ***
Male	63 (12.8)	177 (35.8)	254 (51.4)		
Mode of Delivery, *n* (%)					
CS	70 (10.5) [AR = −3.0]	198 (29.7) [AR = −5.5]	398 (59.8) [AR = 7.2]	0.001 ***	0.001 ***
NSVD	38 (18.3) [AR = 3.0]	105 (50.5) [AR = 5.5]	65 (31.3) [AR = −7.2]		

Mean ± standard deviation, n (%). Median (Q1–Q3). BW: Birth weight; CS: cesarean section; GA: gestational age; NSVD: Normal Spontaneous Vaginal Delivery. AR: Adjusted residual; |AR| > 1.96 indicates statistical significance (*p* < 0.05). Post hoc comparisons were evaluated using the adjusted residual in Mode of Delivery. * Kruskal–Wallis test; post hoc comparisons evaluated using Dunn test with Bonferroni correction in GA (Group 1–2 *p* < 0.05, Group 1–3 *p* > 0.05, Group 2–3 *p* < 0.05). ** ANOVA. *** Chi-Square. ^#^ Mann–Whitney U test. ^##^ Independent Sample *t* test.

**Table 2:** Comparison of hematological and biochemical parameters according to sepsis status and microbiological subgroup.

	Sepsis (*n* = 411)		Non-Sepsis (*n* = 463)		
Variables	Gram Negative (*n* = 108) Group 1	Gram Positive (*n* = 303) Group 2	Control (*n* = 463) Group 3	*p* ^1^ (Three Group Comparisons)	*p* ^2^ (Sepsis–Non- Sepsis)
WBC (×10^3^/µL)	10.9 (6.9–15.5) ^a^	12.1 (8.8–15.9) ^ab^	12.2 (10.2–15) ^b^	0.009	0.041
RBC (×10^6^/µL)	3.8 (3.3–4.3) ^a^	4.0 (3.6–4.7) ^b^	4.4 (4.0–4.8) ^c^	0.001	0.001
Hb (g/dL)	12.8 (10.6–15.7) ^a^	14.5 (12.4–16.4) ^b^	15.4 (14.2–16.8) ^c^	0.001	0.001
RDW (%)	16.3 (15.5–18.2) ^a^	16.8 (15.8–17.9) ^a^	16.5 (15.7–17.5) ^ab^	0.054	0.040
PLT (×10^3^/µL)	192 (94–318) ^a^	270 (183–360) ^b^	268 (217–333) ^b^	0.001	0.001
Albumin (g/dL)	3.1 (2.7–3.5) ^a^	3.4 (3.0–3.7) ^b^	3.5 (3.4–3.7) ^c^	0.001	0.001
CRP (mg/dL)	2.95 (0.79–6.55) ^a^	1.40 (0.70–2.90) ^a^	0.04 (0.02–0.12) ^b^	0.001	0.001
RAR	5.40 (4.69–6.87) ^a^	5.03 (4.41–5.67) ^b^	4.66 (4.29–5.09) ^c^	0.001	0.001
CAR	0.87 (0.25–2.22) ^a^	0.41 (0.20–0.80) ^a^	0.01 (0.006–0.033) ^b^	0.001	0.001
NLR	2.18 (1.16–3.98) ^a^	1.58 (0.82–3.31) ^b^	1.38 (0.90–2.03) ^c^	0.001	0.001
PAR	61,856.3 (32,500.0–98,589.3) ^a^	80,232.6 (53,055.6–107,500.0) ^b^	75,277.8 (62,564.1–94,594.6) ^b^	0.008	0.252
PLR	75.99 (36.06–118.07) ^ab^	72.70 (46.72–113.95) ^a^	65.33 (51.56–82.33) ^b^	0.025	0.007

Data are presented as median Q1–Q3. *p* ^1^ values were obtained from three group comparisons using the Kruskal–Wallis test. Post hoc pairwise comparisons were performed using Dunn’s test with Bonferroni correction. Groups not sharing the same superscript letter differ significantly from each other. *p* ^2^: Sepsis vs. non-sepsis *p*-values were obtained from two group comparisons using the Mann–Whitney U test. CAR: CRP-to-albumin ratio; CRP: C-reactive protein; Hb: hemoglobin; NLR: neutrophil-to-lymphocyte ratio; PAR: platelet-to-albumin ratio; PLR: platelet-to-lymphocyte ratio; PLT: platelet count; RAR: RDW-to-albumin ratio; RBC: red blood cell count; RDW: red cell distribution width; WBC: white blood cell count.

**Table 3:** Diagnostic accuracy of inflammatory and hematological biomarkers for the diagnosis of neonatal sepsis.

Variables	AUC	95% CI	Cutoff	Sn %	Sp %	Youden Index	PPV	NPV	LR+	LR−
RAR	0.647	[0.61–0.69]	5.108	50.7	76.7	0.28	65.8	63.8	2.18	0.64
CAR	0.981	[0.97–0.99]	0.107	88.5	98.7	0.87	98.4	90.7	68.47	0.12
NLR	0.592	[0.55–0.63]	2.703	36.1	86.9	0.23	70.8	60.6	2.75	0.74
CRP	0.978	[0.97–0.99]	0.395	88.3	99.6	0.88	99.5	90.6	204.84	0.12
Albumin *	0.674	[0.64–0.71]	≤3.25	47.3	84.7	0.32	73.2	64.5	3.09	0.62

AUC: Area under the receiver operating characteristic curve; CAR: C-reactive protein-to-albumin ratio; CI: confidence interval; CRP: C-reactive protein; LR+: positive likelihood ratio; LR−: negative likelihood ratio; NLR: neutrophil-to-lymphocyte ratio; NPV: negative predictive value; PPV: positive predictive value; RAR: red cell distribution width-to-albumin ratio; Sn %: sensitivity; Sp %: specificity. *: For albumin and hemoglobin, lower values were associated with neonatal sepsis; therefore, diagnostic thresholds were interpreted in the reverse direction.

**Table 4:** Multivariable logistic regression analysis of factors associated with culture-confirmed neonatal sepsis.

Variables	β	SE	aOR	95% CI for OR	*p*
RAR	0.784	0.089	2.190	1.838–2.608	<0.001
GA, weeks	0.240	0.076	1.271	1.096–1.474	0.002
BW, g	−0.017	0.016	0.983	0.952–1.016	0.309
Sex, male vs. female	0.147	0.152	1.158	0.860–1.561	0.334
Mode of delivery, CS vs. NSVD	−1.278	0.186	0.278	0.193–0.401	<0.001

Note: The dependent variable was sepsis (1 = sepsis, 0 = non-sepsis/control). The model included RAR, gestational age, birth weight (per 100-g increase), sex, and mode of delivery. Female sex and NSVD were used as the reference categories. The overall model was statistically significant (likelihood-ratio χ^2^(5) = 167.709, *p* < 0.001), with −2 log likelihood = 1040.574, Cox–Snell R^2^ = 0.175, and Nagelkerke R^2^ = 0.233. The Hosmer–Lemeshow goodness-of-fit test indicated adequate model fit (χ^2^(8) = 2.427, *p* = 0.965). Overall classification accuracy was 68.5% at a probability cut-off of 0.50. β: Regression coefficient; aOR: adjusted odds ratio; BW: birth weight; CI: confidence interval; CS: cesarean section; GA: gestational age; NSVD: normal spontaneous vaginal delivery; RAR: red cell distribution width-to-albumin ratio; SE: standard error.

## Data Availability

Due to hospital policies, patient data and study materials cannot be shared. However, the dataset is available from the corresponding author upon reasonable request.

## Abbreviations

AUC	Area Under the Curve
BW	Birth Weight
CAR	CRP-to-Albumin Ratio
CoNS	Coagulase-Negative Staphylococci
CRP	C-Reactive Protein
CS	Cesarean Section
GA	Gestational Age
Hb	Hemoglobin
Htc	Hematocrit
NICU	Neonatal Intensive Care Unit
NLR	Neutrophil-to-Lymphocyte Ratio
NSVD	Normal Spontaneous Vaginal Delivery
PAR	Platelet-to-Albumin Ratio
PICU	Pediatric Intensive Care Unit
PLR	Platelet-to-Lymphocyte Ratio
PLT	Platelet
RAR	RDW-to-Albumin Ratio
RBC	Red Blood Cell Count
RDW	Red Cell Distribution Width
ROC	Receiver Operating Characteristic
WBC	White Blood Cell Count

## References

[1] Strunk T., Molloy E.J., Mishra A., Bhutta Z.A. (2024). Neonatal bacterial sepsis. Lancet.

[2] Celik I.H., Hanna M., Canpolat F.E., Pammi M. (2022). Diagnosis of neonatal sepsis: The past, present and future. Pediatr. Res..

[3] Martin S.L., Desai S., Nanavati R., Colah R.B., Ghosh K., Mukherjee M.B. (2019). Red cell distribution width and its association with mortality in neonatal sepsis. J. Matern. Fetal Neonatal Med..

[4] Młodawska M., Młodawski J., Świercz G., Zieliński R. (2022). The Relationship between Nuchal Cord and Adverse Obstetric and Neonatal Outcomes: Retrospective Cohort Study. Pediatr. Rep..

[5] Kumar J., Soni P.K., Angrup A., Saini S.S., Sundaram V., Mukhopadhyay K., Dutta S., Kumar P. (2023). Antimicrobial Resistance Patterns Among Neonates Referred to Pediatric Emergency in North India: A Prospective Cohort Study. Pediatr. Infect. Dis. J..

[6] Li T., Li X., Wei Y., Dong G., Yang J., Yang J., Fang P., Qi M. (2021). Predictive Value of C-Reactive Protein-to-Albumin Ratio for Neonatal Sepsis. J. Inflamm. Res..

[7] Cantey J.B., Lee J.H. (2021). Biomarkers for the Diagnosis of Neonatal Sepsis. Clin. Perinatol..

[8] Ramavath C., Katam S.K., Vardhelli V., Deshabhotla S., Oleti T.P. (2023). Examining the Utility of Rapid Salivary C-Reactive Protein as a Predictor for Neonatal Sepsis: An Analytical Cross-Sectional Pilot Study. Diagnostics.

[9] Wang X., Shi L., Wang C., Ma X. (2023). Therapeutic hypothermia can cause non-infective C-reactive protein elevating. Front. Pediatr..

[10] Eichberger J., Resch E., Resch B. (2022). Diagnosis of Neonatal Sepsis: The Role of Inflammatory Markers. Front. Pediatr..

[11] Mouliou D.S. (2023). C-Reactive Protein: Pathophysiology, Diagnosis, False Test Results and a Novel Diagnostic Algorithm for Clinicians. Diseases.

[12] Xu W., Huo J., Chen G., Yang K., Huang Z., Peng L., Xu J., Jiang J. (2022). Association between red blood cell distribution width to albumin ratio and prognosis of patients with sepsis: A retrospective cohort study. Front. Nutr..

[13] Ma C., Liang G., Wang B., Eisenhut M., Urrechaga E., Wiedermann C.J., Andaluz-Ojeda D., O’Rourke J., Zhang Z., Jin X. (2024). Clinical value of the red blood cell distribution width to albumin ratio in the assessment of prognosis in critically ill patients with sepsis: A retrospective analysis. J. Thorac. Dis..

[14] Yin W., Fang C., Fan X., Chen Y. (2024). Albumin and C-reactive protein as diagnostic markers for neonatal sepsis: A retrospective study. J. Int. Med. Res..

[15] Yang C., Yang Y., Li B., Xu P., Shen Q., Yang Q. (2016). The diagnostic value of high-sensitivity C-reactive protein/albumin ratio in evaluating early-onset infection in premature. Zhonghua Wei Zhong Bing Ji Jiu Yi Xue.

[16] Güneş H., Yurttutan S., Çobanuşağı M., Doğaner A. (2021). CRP/albumin ratio: A promising marker of gram-negative bacteremia in late-onset neonatal sepsis. Turk. Arch. Pediatr..

[17] Dogan P., Guney Varal I. (2020). Red cell distribution width as a predictor of late-onset Gram-negative sepsis. Pediatr. Int..

[18] Bulut O., Akcakaya A., Bulut N., Ovali F. (2021). Elevated Red Cell Distribution Width as a Useful Marker in Neonatal Sepsis. J. Pediatr. Hematol. Oncol..

[19] Liu Z.Y., Jiang H.Z., Wang L., Chen M.X., Wang H.T., Zhang J.X. (2022). Diagnostic accuracy of red blood cell distribution width for neonatal sepsis. Minerva Pediatr..

[20] Xu Y., Qi W. (2023). Association between red cell distribution width to albumin ratio and acute kidney injury in patients with sepsis: A MIMIC population-based study. Int. Urol. Nephrol..

[21] Shan X., Jiang J., Li W., Dong L. (2024). Red blood cell distribution width to albumin ratio as a predictor of mortality in ICU patients with community acquired bacteremia. Sci. Rep..

[22] Hong J., Hu X., Liu W., Qian X., Jiang F., Xu Z., Shen F., Zhu H. (2022). Impact of red cell distribution width and red cell distribution width/albumin ratio on all-cause mortality in patients with type 2 diabetes and foot ulcers: A retrospective cohort study. Cardiovasc. Diabetol..

[23] Huang M., Liu F., Li Z., Liu Y., Su J., Ma M., He Y., Bu H., Gao S., Wang H. (2023). Relationship between red cell distribution width/albumin ratio and carotid plaque in different glucose metabolic states in patients with coronary heart disease: A RCSCD-TCM study in China. Cardiovasc. Diabetol..

[24] Jing R., Yu B., Xu C., Zhao Y., Cao H., He W., Wang H. (2024). Association between red cell distribution width-to-albumin ratio and prognostic outcomes in pediatric intensive care unit patients: A retrospective cohort study. Front. Pediatr..

[25] Vandenbroucke J.P., von Elm E., Altman D.G., Gøtzsche P.C., Mulrow C.D., Pocock S.J., Poole C., Schlesselman J.J., Egger M., STROBE Initiative (2007). Strengthening the Reporting of Observational Studies in Epidemiology (STROBE): Explanation and elaboration. PLoS Med..

[26] Hu Z.D., Lippi G., Montagnana M. (2020). Diagnostic and prognostic value of red blood cell distribution width in sepsis: A narrative review. Clin. Biochem..

[27] Wang T.H., Hsu Y.C. (2021). Red Cell Distribution Width as a Prognostic Factor and Its Comparison with Lactate in Patients with Sepsis. Diagnostics.

[28] Mlodawska M., Zmelonek-Znamirowska A., Swiercz G., Mehta J., Mlodawski J. (2026). Neonatal Respiratory Outcomes in Placenta Previa with Histopathologically Confirmed Placenta Accreta Spectrum. J. Clin. Med..

[29] Örnek Z. (2026). Association Between the Red Cell Distribution Width-to-Albumin Ratio and Pediatric Community-Acquired Pneumonia. Pediatr. Int..

